# SERS Properties of Gold Nanorods at Resonance with Molecular, Transverse, and Longitudinal Plasmon Excitations

**DOI:** 10.1007/s11468-014-9669-4

**Published:** 2014-03-07

**Authors:** Ida Ros, Tiziana Placido, Vincenzo Amendola, Chiara Marinzi, Norberto Manfredi, Roberto Comparelli, Marinella Striccoli, Angela Agostiano, Alessandro Abbotto, Danilo Pedron, Roberto Pilot, Renato Bozio

**Affiliations:** 1 Department of Chemical Sciences, University of Padova, Via Marzolo 1, 35131 Padova, Italy; 2 INSTM, University of Padova, Via Marzolo 1, 35131 Padova, Italy; 3 Department of Chemistry, University of Bari, Via Orabona 4, Bari, 70126 Italy; 4 Department of Materials Science and INSTM, University of Milano-Bicocca, Via Cozzi 53, 20125 Milano, Italy; 5 CNR-IPCF Division of Bari, c/o Department of Chemistry, University of Bari, Via Orabona 4, Bari, 70126 Italy; 6 Department of Chemical Sciences, University of Padova and INSTM, Via Marzolo 1, 35131 Padova, Italy

**Keywords:** Gold nanorods, Localized surface plasmon resonance, SERS, Nonlinear optics, Push–pull molecule

## Abstract

The amplification of Raman signals of the heteroaromatic cation 1-(N-methylpyrid-4-yl)-2-(N-methylpyrrol-2-yl)ethylene (**PEP+**)) bound to Au nanorods (NRs) was investigated at different excitation wavelengths to study the effect of the laser resonance with the absorption band of the **PEP+** moiety and with the two plasmon oscillation modes of the NR. Two different **PEP+** derivatives, differing in the length of the alkyl chain bearing the anchoring group, were used as target molecules. Raman spectra obtained exciting at 514 or at 785 nm (i.e., exciting the transverse or the longitudinal plasmon band) present a higher intensity than that at 488 nm suggesting a higher Raman amplification when the laser excitation wavelength is resonant with one of the two plasmon modes. Moreover, considering results of Discrete Dipole Approximation (DDA) calculations of the local field generated at the NR surface when either the transverse or the longitudinal plasmon modes are excited, we deduced that the resonance condition of the 514-nm laser excitation with the absorption band of the dye strongly contributes to the amplification of the Raman signal.

## Introduction

Raman spectroscopy, owing to its ability to recognize typical molecular fingerprints, provides a unique approach for solving analytical problems [1]. However, Raman scattering has an extremely small cross section, typically 10^−30^ to 10^−25^ cm^2^, which prevents the use of this technique as a method for ultrasensitive trace detection. Raman scattering literally appears in a new light when it takes place in the local optical field of metal nanostructures [2]. These systems, thanks to the ability of sustaining strong localized surface plasmon resonances (LSPR), provide the key effect for the observation of enhanced Raman signals from molecules attached to them (Surface Enhanced Raman Spectroscopy—SERS). The enhancement depends on the type of metal and on the nanostructure features and it is particularly strong when both laser and scattered field are in resonance with the surface plasmons [3].

Several examples of nanostructures of different shape, metal and, possibly, assembled in ordered or random arrangements are reported in the literature, resulting in electromagnetic SERS enhancement factors up to 10^8^ [1, 4–10]. For example, rod-shaped metal nanoparticles possess two plasmon resonances, a transverse mode perpendicular to the long axis of the rod and a longitudinal mode parallel to the long rod axis [11]. The latter depends linearly on the aspect ratio, i.e., the length divided by the width of the nanorods (NRs), and it is widely tunable in the visible and in the near infrared region of the spectrum. In addition, both plasmon resonances depend on the aggregation state of the nanoparticles and on the refractive index of the surrounding medium. The possibility to tune the resonance wavelength can be exploited to amplify both laser and scattered field in SERS measurements at the desired wavelength.

Despite the attractive characteristics of metallic NRs as SERS substrates, only a few reports exist for SERS on NRs where the Raman excitation occurs at a wavelength that overlaps with NRs plasmon resonance [12–18]. To our knowledge, only Orendorff et al. [15] and Guo et al. [17] conducted a shape-dependent SERS study in dilute, not aggregated, colloids of silver and gold NRs, concluding that overlapping the excitation line with the longitudinal SPR contributes an additional enhancement by a factor of 10–10^2^.

**Fig. 1:**
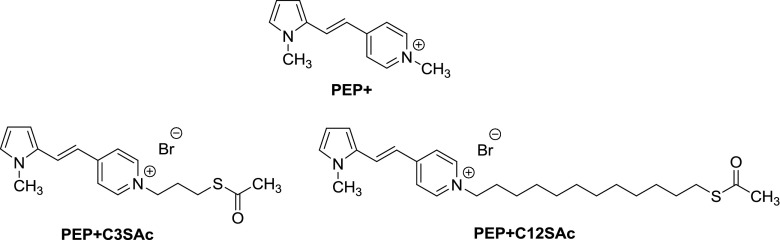
Molecular structures of parent dipolar species **PEP+** and its ω-acetylthioalkyl derivatives **PEP+C3SAc** and **PEP+C12SAc**

In this work, we investigate the amplification of the Raman signals of the heteroaromatic cation 1-(*N*-methylpyrid-4-yl)-2-(*N*-methylpyrrol-2-yl)ethylene (**PEP+**) bound to Au NRs. The dipolar, positively charged dye **PEP+** (Fig. 1) is a push–pull molecule composed by a π-deficient (pyridinium ion) as the acceptor group (A) and a π-excessive (pyrrole) heterocycle as the donor group (D) [19]. **PEP+** and its derivatives present remarkable resonant and non-resonant nonlinear optical properties, such as two-photon absorption and second harmonic generation [20–22]. In particular, we have thoroughly investigated the linear and non-linear optical properties of this class of push-pull systems for application in optical limiting and up-converted lasing devices [23–25]. To improve the hyperpolarizability, and in so doing the nonlinear optical properties, chemists have been modifying the electron affinity of the acceptor and the ionization potential of the donor groups by selecting more or less polar substituents. The amplified local field at the surface of metal nanoparticles is a further possible means of enhancing the effective nonlinear optical responses. A preliminary investigation of the SERS properties of this type of dye opens the way to evaluating the effectiveness of this enhancement since both SERS and two-photon absorption exhibit a similar dependence on the fourth power of the local electric field.

In order to graft the **PEP+** ion on Au NRs, we have synthesized acetylthio terminated **PEP+** chromophores spaced by two linear alkyl chains of different length, containing 3 (**PEP+C3SAc**) or 12 (**PEP+C12SAc**) carbon atoms (Fig. 1).

The basic idea is to control the intensity of the Raman signals as a function of the distance between the dye and the metallic surface of the metal nanostructure and to study the effect of the resonance of laser wavelengths with the absorption band of **PEP+** moiety (molecular resonance) and with the two plasmon modes (plasmonic resonances).

The Raman characterization of NRs functionalized with **PEP+C3SH** and **PEP+C12SH**, both deposited on glass substrates and in solution, is realized using different laser excitation lines. The 488-nm and the 514-nm laser excitation lines are in near resonance with the absorption band of the molecules, yielding surface enhanced resonance raman scattering (SERRS), whereas the 785-nm laser excitation line is off-resonant with molecular excitations. On the other hand, the 514-nm and the 785-nm lines are resonant with the transverse and longitudinal modes of Au NRs, respectively, suggesting a SERS-type enhancement of the Raman signals.

4-Mercaptopyridine bound to NRs is used to calculate the SERS enhancement factor (EF) for the transverse and longitudinal plasmon band excitations. Without possible interference with molecular resonances, the measured EFs provide an experimental probe of the local field. The results turn out to be in fair agreement with the theoretical values obtained by the Discrete Dipole Approximation (DDA) calculation of the local electromagnetic field intensity around a single NR.

## Results and Discussion

### Optical and Morphological Characterization of Bare Au NRs

**Fig. 2:**
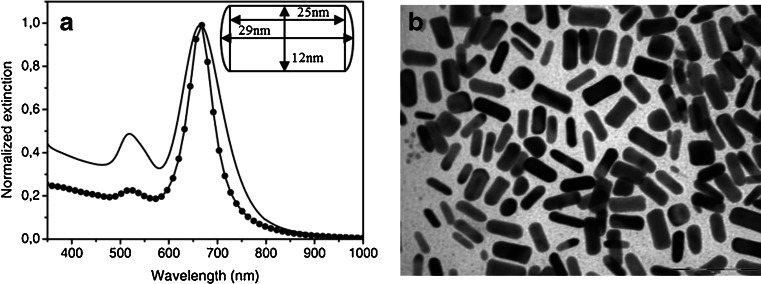
Extinction spectrum (**a**) and relative TEM image (**b**) of water soluble Au NRs prepared by seed mediated method. The *dotted line* corresponds to the extinction spectrum calculated by the DDA method

CTAB-stabilized Au NRs prepared by seed mediated growth method exhibited a transverse and longitudinal plasmon absorption bands at 520 and 670 nm, respectively. The normalized extinction spectrum of CTAB-stabilized Au NRs in water is shown in Fig. 2a, the dotted line corresponds to the orientationally averaged extinction spectrum obtained via the DDA method. The average size of NRs was worked out by averaging more than hundred particles in TEM images (a typical one is reported in Fig. 2b). The average dimensions turn out to be 29 ± 3 nm in length and 12 ± 2 nm in diameter, thus the aspect ratio (length to diameter) is approximately 2.4.

Both experimental and theoretical spectra in Fig. 2 have been normalized to the maximum extinction, that is the peak intensity of the longitudinal plasmon.

Calculated and experimental spectra differ in two features, namely: (i) the different intensity ratio between longitudinal and transverse plasmon and (ii) the larger spectra width of the longitudinal plasmon resonance in the experimental spectrum. Looking at the TEM image of our sample in Fig. 2b, where over 100 particles are imaged, just few roundish/cubic NPs are evident (around 8 %), therefore we find unlikely that (i) arises from the presence of spherical particles whose contribution is superimposed to the transverse plasmon. We rather envision the following explanation for points (i) and (ii):

Polydispersity of our sample generates a distribution of aspect ratios. The longitudinal plasmon resonance is more sensitive to the aspect ratio than the transverse one. This translates into an inhomogenous broadening of the longitudinal plasmon that alters the peak intensity ratio of the experimental spectrum compared to the calculated one.

From a microscopic point of view, the peak width of both longitudinal and transverse mode is influenced also by surface damping effects when the nanoparticle size becomes comparable to the mean free path of Gold (50 nm) [26] therefore, in addition to polydispersity, surface dumping effects as well contribute to the observed spectral width both in the longitudinal and in the transverse peak. However, this effect is accounted for in the calculated spectra since a size corrected and anisotropic dielectric constant is used, as mentioned in the experimental section. Therefore, we would infer that point (ii) is due to the polydispersity of our sample, as hypothesized above.

### Near Field DDA Simulations of Bare Nanorods

**Fig. 3:**
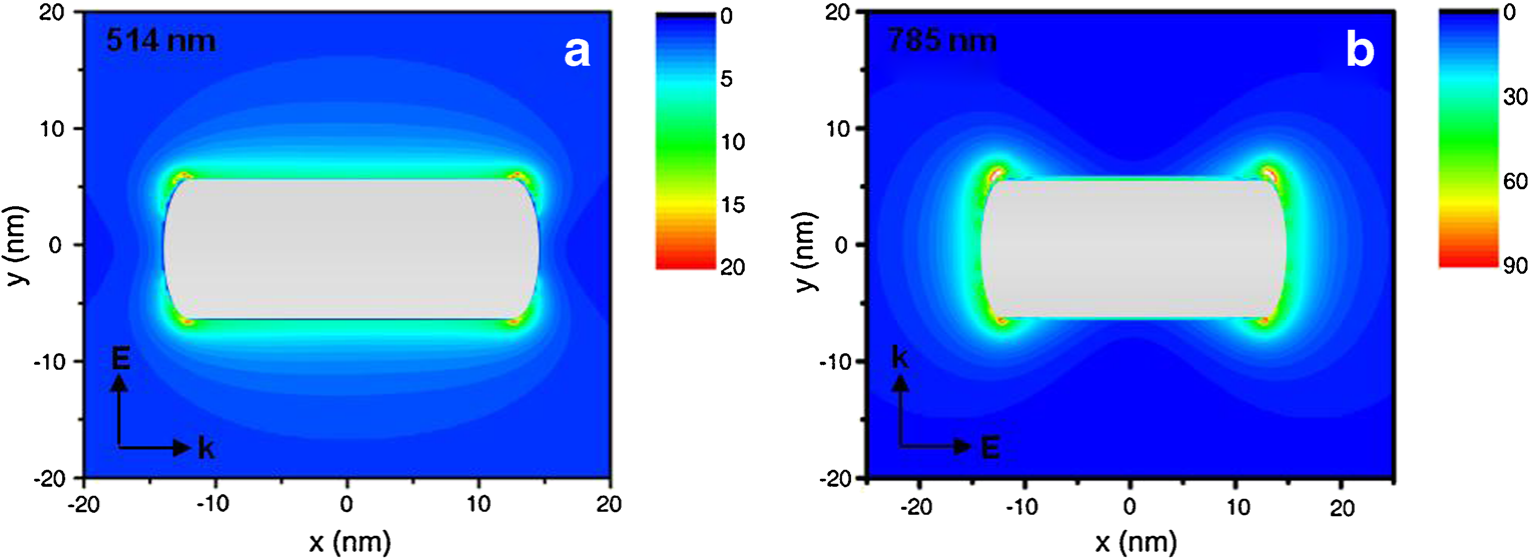
Calculated distribution of the square of the electromagnetic field around a single particle at (**a**) 514 nm for the transverse plasmon and at (**b**) 785 nm for the longitudinal plasmon

DDA calculations on NRs with the geometrical parameters defined in Fig. 2 were carried out and are described in the following. The square of the electric field around the rod is plotted in Fig. 3. As shown, the longitudinal mode (excited at 785 nm) is characterized by an electric field intensity that reaches values about 10 times higher than the transverse one (excited at 514 nm). Moreover, the longitudinal mode is mainly localized at the caps of the rod, whereas the transverse one spreads on the particle sides. Numerically, the SERS enhancement averaged over the whole particle surface, turned out to be about 10^4^ and 10^2^, respectively, therefore corresponding to a longitudinal/transverse ratio of about 100.

### Optical and Morphological Characterization of Functionalized Au NRs

#### IR and TEM

**Fig. 4:**
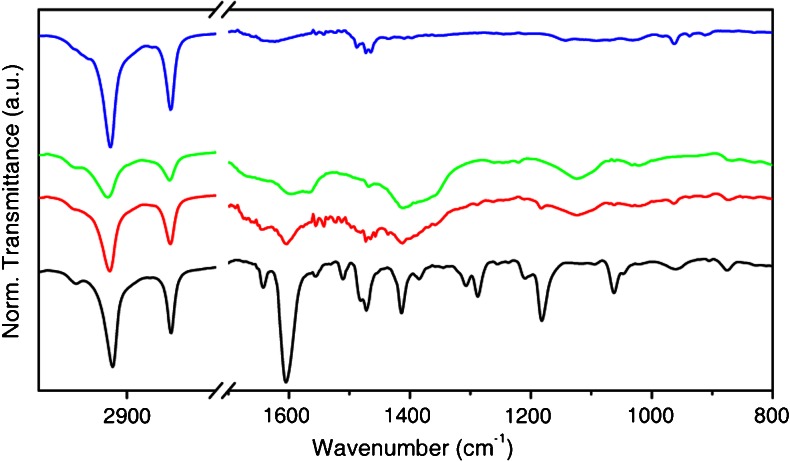
FT-IR spectra of NRs functionalized with **PEP+** moiety: **PEP+C3SH** (*black*), **PEP+C3-NRs** (*red*), **PEP+C12-NRs** (*green*), and CTAB stabilized **NR**s (*blue*)

Infrared spectroscopy of thiol functionalized gold nanostructures is routinely used in order to establish the binding of thiols and to study the structure of the organic ligand shell [27]. Figure 4 shows FT-IR spectra of **PEP+C3SH** (black line), **PEP+C3SH** functionalized Au NRs (**PEP+C3-NRs**, red line), **PEP+C12SH** functionalized Au NRs (**PEP+C12-NRs**, green line), and CTAB stabilized Au NRs (blue line). In the IR spectrum of CTAB stabilized NRs (blue line), the vibrational modes characteristic of CTAB are clearly visible. The methylene C-H stretching modes appear at 2,918 and 2,850 cm^−1^, whereas the alkyl bending vibrations appear at 1,480 cm^−1^. When NRs are functionalized with **PEP+C3SH** (red line) or **PEP+C12SH** (green line), in the IR spectrum new vibrational modes emerge that we associate to the **PEP+** moiety (black line): the C=C aromatic stretching at 1,604 cm^−1^ and the C–N stretching at 1,182 cm^−1^. The frequencies of the signals originating from C–H stretching modes have been often used to establish the degree of order and the extent to which the all-trans conformation is adopted by aliphatic chains in self-assembled monolayers on Au flat surface and nanoparticles [27, 28]. For example, NP coated with alkanethiols containing six or more methylene groups in the chain present the methylene C–H symmetric stretching mode at 2,850 cm^−1^, characteristic of an all-trans conformation of the alkyl chains. Instead, shorter alkanethiols coating usually present several gauche defects, observable through a shift of this mode signal to higher frequency.

The methylene C–H symmetric stretching mode is at 2,850 cm^−1^ for both **PEP+C3-NRs** and **PEP+C12-NRs**, consistent with an all-trans conformation of the alkyl chains in these systems. This finding suggests that, in both cases, the alkyl linkers between the thiol group and the **PEP+** moieties are fully extended. Therefore, the distance between the **PEP+** moiety and the surface-anchoring thiol group is ca. 1.6 nm for **PEP+C12SH** and ca. 0.5 nm for **PEP+C3SH**.

#### UV–VIS

**Fig. 5:**
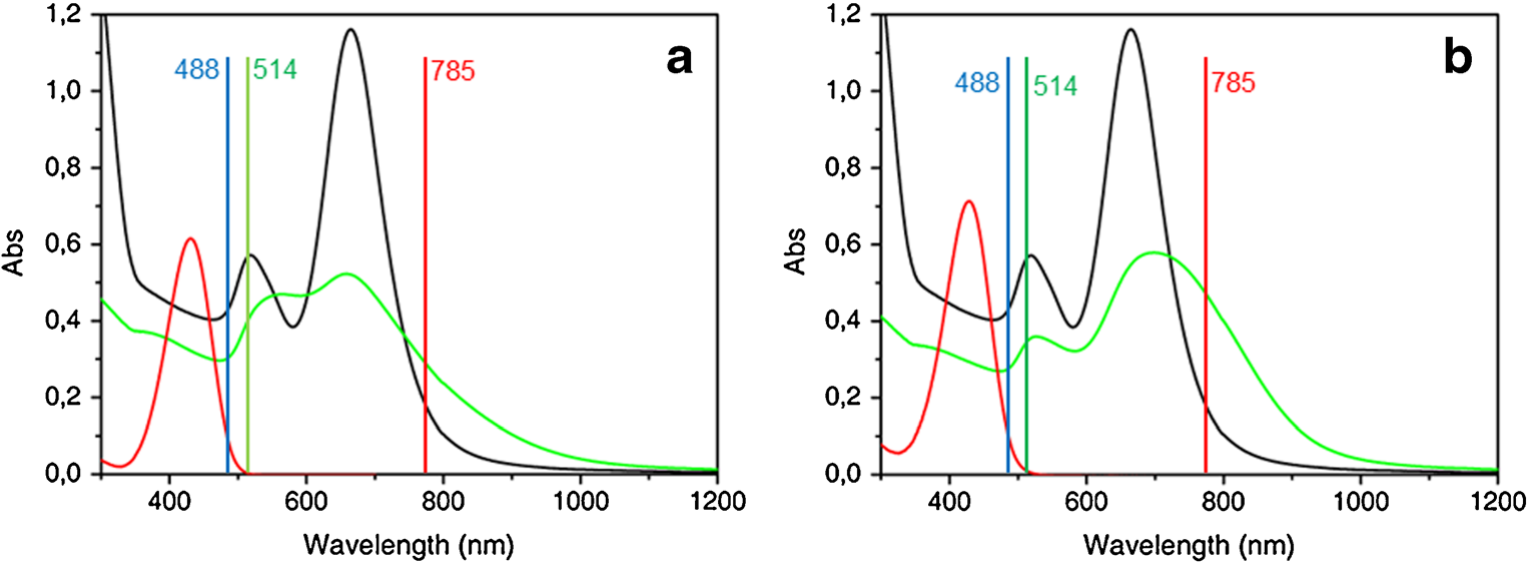
UV-visible absorption spectra of bare NRs in water (*black trace*), dye (*red trace*), and dye functionalized NRs (*green trace*) for the case of **PEP+C3SH** (**a**) and for **PEP+C12SH** (**b**)

In Fig. 5a and b, the UV–visible extinction spectra of Au NRs before and after the functionalization procedure with **PEP+C3SH** and **PEP+C12SH** are shown. Both water soluble **PEP+C3SH** and **PEP+C12SH** (red traces in Fig. 5a and b) exhibit a narrow absorption band with a maximum at 431 and at 428 nm, respectively.

After the functionalization process, both longitudinal and transverse plasmon absorption features are retained for NRs despite repeated washing by centrifugation/re-dispersion cycles (up to 10 times) to remove free dye molecules in solution. Interestingly, performing the same procedure on CTAB-stabilized Au NRs causes the nanoparticles to aggregate as suggested by the flattening and broadening of the longitudinal plasmon band in the UV-visible spectrum (data not shown). This means that the **PEP+** functionalization somehow prevents NRs aggregation. A slight broadening of longitudinal and transverse plasmon absorption bands is clearly visible for Au NRs functionalized with **PEP+C3SH** (**PEP+C3-NRs**) and **PEP+C12SH** (**PEP+C12-NRs**). In addition, a 7-nm blue shift and a 30-nm red shift in the longitudinal peak maximum can be observed in the green traces of Fig. 5a and b, respectively. The interaction of Au NRs with thiol groups typically results in a dampening and a broadening of the plasmon absorption bands, as described in the literature [11, 29]. Furthermore, it should be emphasized that replacing CTAB with either **PEP+C3SH** or **PEP+C12SH**, i.e., with heterocyclic dyes possessing large nonlinear responses, implies a strong increase in the polarizability of the capping layer and contributes to the shift of the plasmon resonances.

**PEP+C3-NR** and **PEP+C12-NR** extinction spectrum shows in both cases a weak shoulder around 400 nm, ascribable to the dye, slightly blue shifted with respect to the absorption band of the free **PEP+** moiety. Despite the difficulty in unequivocally defining the band position of the dye after binding to Au NRs, due to the low absorbance value, the apparent blue shift of the band suggests that the adsorbed dye molecules interact with each other like they do in H-type aggregates of PEP+ commonly reported in the literature [20, 21]. Another possible origin of the changes in the molecular resonance may arise from some degree of hybridization between molecular and plasmon excitations in cases in which the two are close to coincidence. Such phenomenon has been recently analyzed theoretically [30].

**Fig. 6:**
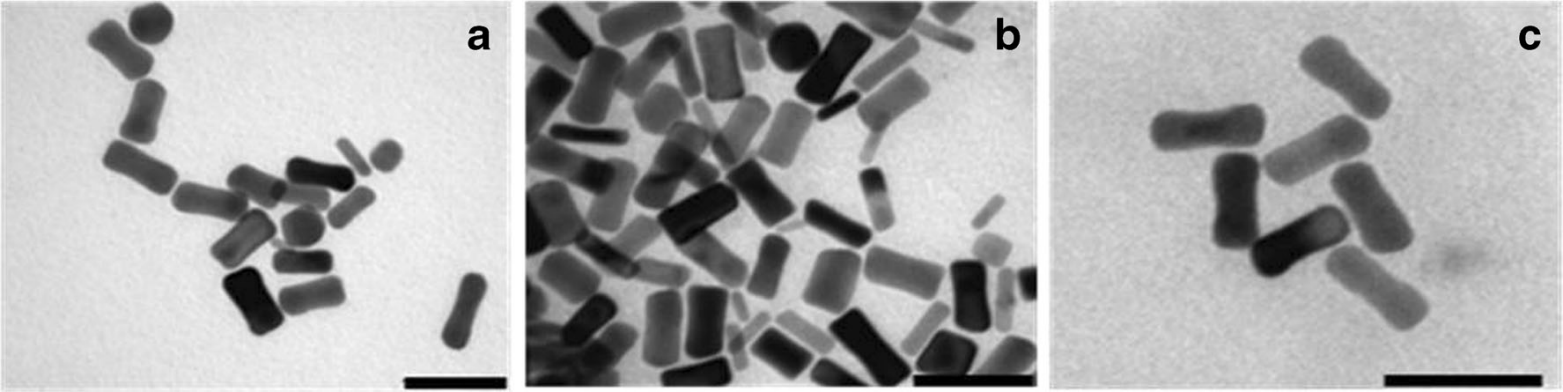
TEM images of (**a**) Au NRs, (**b**) **PEP+C3-NRs**, and (**c**) **PEP+C12-NRs** purified by dye excess at 12 h (*scale bar* 50 nm)

The TEM images of the Au NRs in water before functionalization (Fig. 6a) and after the anchoring of **PEP+C3SH** (Fig. 6b) and **PEP+C12SH** (Fig. 6c) further confirm that no morphological changes occur after binding. The **PEP**-functionalized Au NRs are well separated and exhibit minimal changes in shape and size.

#### Surface Enhanced Raman Spectra of PEP+C3SH and PEP+C12SH Bound to NRs and Deposited on a Glass Slide

Here we aim to the identification and assignment of the Raman bands of **PEP+** in powders and attached to NRs: we worked on solid samples since the Raman signal is much stronger than in the measurements in solution described in the next section.

Due to the **PEP+** fluorescence, spectra of dye powders could be collected only at 785 nm. Spectra at several wavelengths (785, 514, and 488 nm) were collected from NRs functionalized with **PEP+C3SH** and **PEP+C12SH** deposited on a glass slide by drop casting. As shown in Fig. 5, the 488-nm and the 514-nm laser excitation lines are close to resonance with the absorption band of the molecules. Therefore, we can refer to these spectra as surface enhanced resonance Raman spectra (SERRS). When the laser excitation is not in electronic resonance with the molecular system, as for the 785-nm laser excitation line, we can refer to the observed enhanced spectra as surface enhanced Raman spectra (SERS).

**Fig. 7:**
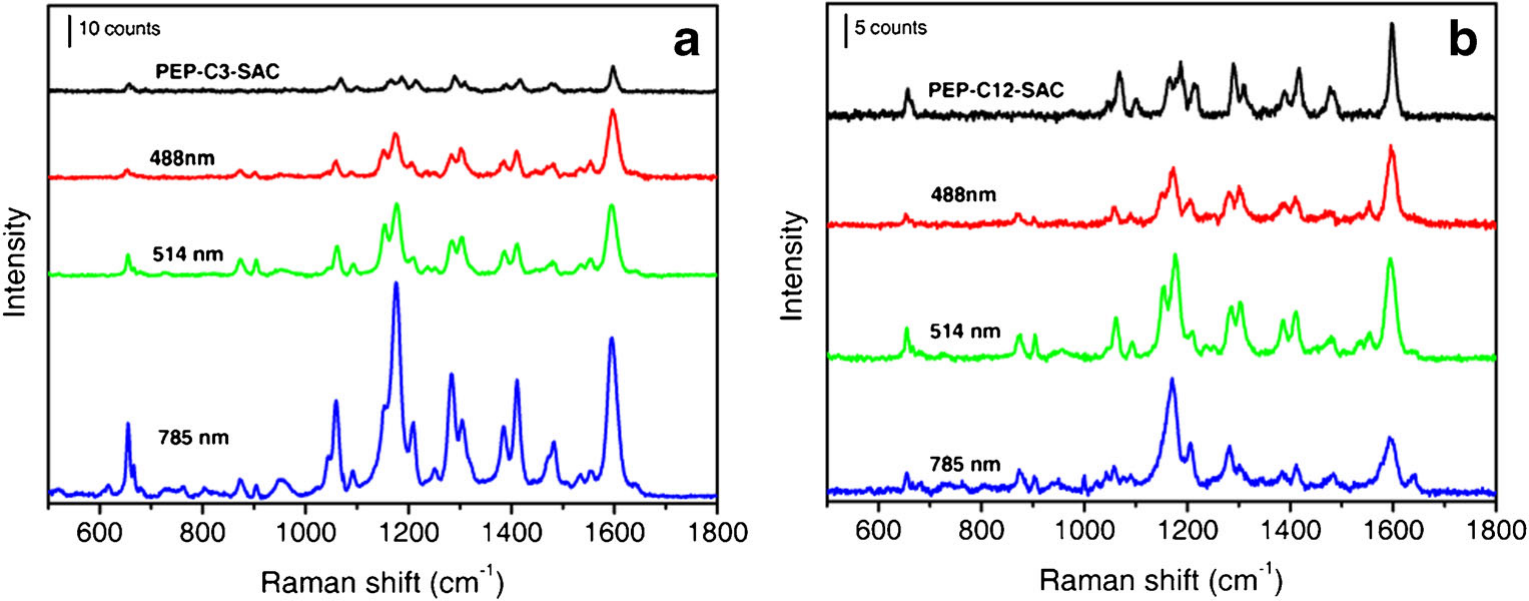
**a** SERRS and SERS spectra of **PEP+C3SH** powders (*black*) and bound to NRs (deposited on a glass slide) at different laser excitation. **b** SERRS and SERS spectra of **PEP+C12SH** powders (*black*) and bound to NRs (deposited on a glass slide) at different laser excitation

Peak frequencies and assignment associated to the pyridinium and the pyrrole groups are given in Table 1 following the literature [31–34]. Figure 7 shows typical spectra (averaged over 5 points) of the powders of the **PEP+** dyes and of the functionalized NRs deposited on a glass slide, normalized with respect to cyclohexane. If we reasonably assume that the number of molecules probed in the dye powders is higher than in **PEP+C3SH-NR** and **PEP+C12SH-NR** ones, the spectra evidence a remarkable SERS effect at 785 nm.

**Table 1:** Peak frequency assignments for neat **PEP+C3SH** and **PEP+C12SH** and for **PEP+C3-NRs** and **PEP+C12-NRs** at different excitation wavelengths (in nm) [31–34]

PEP+C3SH	PEP+C12SH	PEP+C3-NRs			NR PEP+C12-NRs			Assignments
λ_ex_ 785 (cm^−1^)	λ_ex_ 785 (cm^−1^)	λ_ex_ 488 (cm^−1^)	λ_ex_ 514 (cm^−1^)	λ_ex_ 785 (cm^−1^)	λ_ex_ 488 (cm^−1^)	λ_ex_ 514 (cm^−1^)	λ_ex_ 785 (cm^−1^)	
658 and 664	657	654	655 and 667	655 and 667	652 and 665	655 and 667	655 and 666	Ring CH out-of-plane def and bend.
		873 and 901	874 and 904	874 and 904	873 and 901	876 and 904	873 and 902	N=C-H and N–C–H def
1,069	1,068	1,060	1,061	1,060	1,060	1,061	1,058	Ring CH in-plane bend (pyridinium)
1,167, 1,187, 1,214	1,166, 1,187, 1,213	1,152, 1,176, 1,206	1,154, 1,177, 1,209	1,153, 1,176, 1,210	1,150, 1,173, 1,205	1,155, 1,177, 1,212	1,171, 1,206	Ring CH in-plane bend (pyridinium)
1,290 and 1,310	1,290 and 1,310	1,283 and 1,302	1,285 and 1,305	1,283 and 1,304	1,281 and 1,300	1,286 and 1,302	1,282	N–C ring str (pyrrole)
1,390 and 1,417	1,389 and 1,418	1,385 and 1,410	1,387 and 1,412	1,386 and 1,411	1,389 and 1,410	1,387 and 1,412	1,384 and 1,412	Ring vib (pyrrole)
1,478	1,477	1,481	1,480	1,483	1,478	1,480	1,484	CH3 Def in NCH3 (pyrrole)
		1,533 and 1,554	1,535 and 1,554	1,535 and 1,555	1,554	1,535 and 1,585		C=C and C=N ring stretch (pyridinium)
1,597	1,597	1,597	1,595	1,595	1,595	1,595	1,595	C=C and C=N ring stretch (pyridinium)

#### Surface Enhanced Raman and Resonance Raman Spectra of PEP+C3SH and PEP+C12SH Bound to NRs in Solution

SERS/SERRS experiments were repeated on functionalized NRs in aqueous solutions to study the properties of an ensemble of non-interacting nanoparticles. In order to ascertain whether significant NR aggregation occurs in solution, we performed dynamic light scattering (DLS) measurements. The particle size, measured by DLS, was 33 ± 5 nm for bare NRs, close to the long axis length of the NRs measured by TEM. For functionalized NRs the rather poor quality of the DLS measurements does not allow us to rule out the possible presence of limited amounts of dimers. However, the results suggest that extensive aggregation did not take place. Therefore, our SERRS/SERS spectra should not be significantly affected by aggregation or plasmon coupling effects and comparison with DDA simulations of isolated NR is meaningful.

**Fig. 8:**
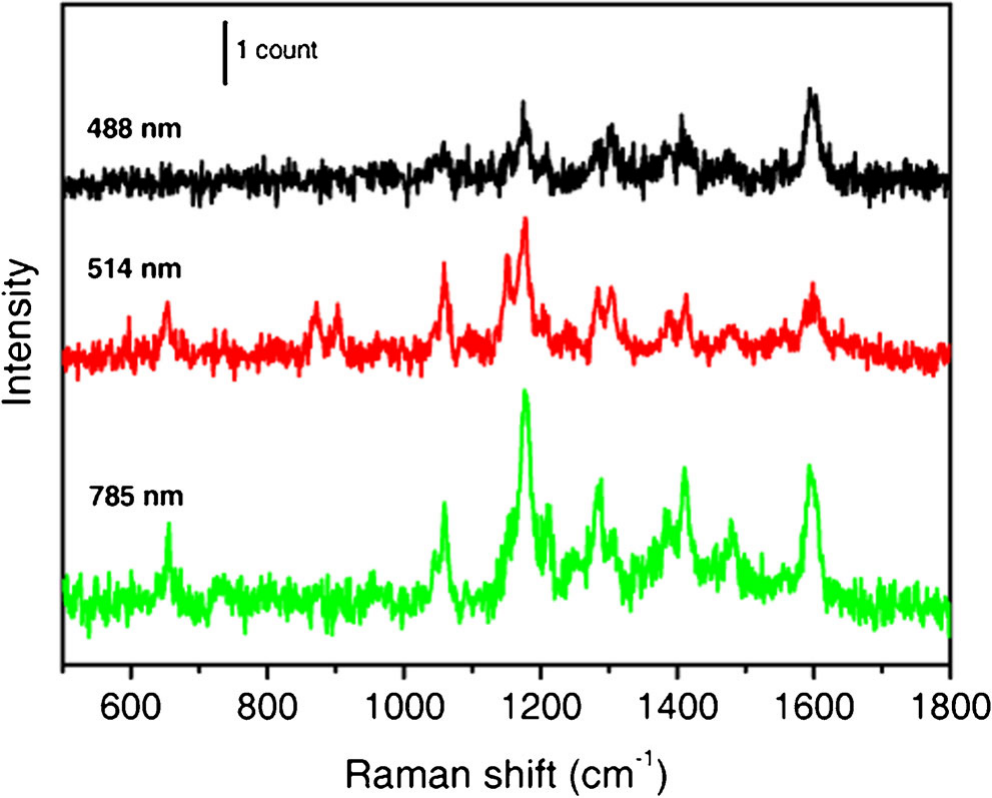
SERRS and SERS spectra, in solution, of **PEP+C3-NRs** at different laser excitation

SERS enhancement factors (EF) can be evaluated by comparing the intensity of a selected Raman band of **PEP+C3SH** bound to **NR** with the intensity of the same band when the dye is free in solution, upon normalization by the number of illuminated molecules. Although, thanks to fluorescence quenching, SERS spectra of **PEP+C3SH-NR** are measurable at all excitation wavelengths used (see spectra in Fig. 8), fluorescence hindered the acquisition of **PEP+C3SH** spectra in solution at 514 and 488 nm. Therefore the procedure above could be used to determine the EF only at 785 nm excitation: details of the method are provided in the following. Similar considerations apply to **PEP+C12SH-NR**.

EFs are obtained by using the expression [15]:

1 $$ EF=\frac{I_{SERS}}{I_{Raman}}\times \frac{M_{PEP+}}{M_{NR- PEP+}} $$

where *M*_*PEP+*_ is the concentration of **PEP+C3SH** molecules sampled in the reference solution, *M*_*NR-PEP+*_ is the concentration of **PEP+C3SH** or **PEP+C12SH** molecules bound to NRs in water solution, *I*_*SERS*_ is the intensity of a vibrational mode in the surface-enhanced spectrum, and *I*_*Raman*_ is the intensity of the same mode in the Raman spectrum. Vibrational modes used to determine enhancement factors include the ring CH out-of-plane deformation and bending (655 cm^−1^) and the in-plane bending (1,069 cm^−1^ and 1,180 cm^−1^) modes, the N-C ring stretching mode (1,289 cm^−1^), the ring vibration mode (1,415 cm^−1^).

*M*_*NR-PEP+*_ has been evaluated by determining the concentration of NRs in solution and the number of PEP+ molecules adsorbed per NR. To evaluate the concentration of NRs in solution, we considered the extinction coefficients calculated by Orendorff et al. [35] and related to the corresponding NRs aspect ratio and longitudinal plasmon peak maximum wavelength. The extinction coefficient (*ε*_*NR*_) at the peak of the longitudinal plasmon band increases linearly with increasing the wavelength (*λ*_*long*_) of the longitudinal plasmon band [35]. From interpolation of the reported data, the following empirical relation can be derived:

2 $$ {\varepsilon}_{NR}\left(\times {10}^9{M}^{-1}c{m}^{-1}\right)=-5+0.012{\lambda}_{long}(nm) $$

Since our NRs present a longitudinal plasmon band at *λ*_*long*_ = 670 nm, the calculated extinction coefficient *ε*_*NR*_ is 3 × 10^9^ M^−1^ cm^−1^. Using the Lambert–Beer law, we calculated NRs concentration (*C*_*NR*_) in water as 2 × 10^12^ particles/mL. The Surface area of a single NR is calculated as *SA*_*NR*_ = 2*π*(*r* ^2^ + *h* ^2^) + 2*πrl* = 1,193 nm^2^ = 1.09 × 10^−15^ m^2^, where *r* = 6 nm is the radius, *l* = 25 nm and *h* = 2 nm (see inset in Fig. 2a). To calculate the number of molecules bound to a single NR, we considered the area occupied by a single molecule [36]. The footprint of a **PEP+** molecule lying edge-on is on the order of 0.5 nm^2^ [21]. If we consider a uniform binding of the thiol on all the surface, the number of molecules for a single NR is given by the ratio of the surface area of a NR to the footprint of the thiol *SA*_*NR*_ */SA*_*thiol*_, which is about 2400. Finally, *M*_*NR-PEP+*_ turns out to be about 7 · 10^− 6^ and EF(785) is in the order of 10^4^ for both **PEP+C3SH-NR** and **PEP+C12SH-NR**.

Concerning the other two excitation wavelengths (488 and 514 nm), we normalized the data, along with those at 785 nm, with respect to cyclohexane bands lying within 30 cm^−1^ of the **PEP+C3-NR** bands: by this method, the ω^4^ dependence is accounted for as well as instrument sensitivity issues. Clearly, on comparing normalized intensities at different excitation wavelengths, the molecular resonance effects do not cancel out.

A parameter *R*, which corresponds to the ratio $\frac{ EF(785)}{ EF\left(488,514\right)}$ except for molecular resonance effects, can be defined:

$$ R\left(488,514\right)=\frac{I^{\mathrm{PEP}+\mathrm{C}3\mathrm{SH}-\mathrm{NR}}(785)}{I^{\mathrm{Cyclohexane}}(785)}\frac{I^{\mathrm{Cyclohexane}}\left(488,514\right)}{I^{\mathrm{PEP}+\mathrm{C}3\mathrm{SH}-\mathrm{NR}}\left(488,514\right)} $$

The results at 488, 514, and 785 nm are summarized in Table 2.

**Table 2:** EF and *R* factors of **PEP+C3SH** and **PEP+C12SH** on Au NRs for different vibrational modes

Band (cm^−1^)	PEP+C3-NR		PEP+C12-NR		4-Mpy-NR
	R (488)	R (514)	EF (785)		EF (785)
655			1.6 × 10^4^	1.4 × 10^4^	
1,069	3.7	3.2	1.1 × 10^4^	1.9 × 10^4^	
1,180	3.0	3.9	1.7 × 10^4^	9.3 × 10^3^	
1,289	2.7	4.3	1.0 × 10^4^		
1,415	4.8	7.2	9.0 × 10^3^		
1,004					3.7 × 10^4^
1,119					1.1 × 10^5^

The values of *R* in Table 2 indicate that the normalized intensities at 785 nm are about 3–7 times higher than the normalized intensities at 514 or 488 nm excitation.

We attempted to perform SERS measurements with a non-resonant probe, that is 4-mercapto-pyridine (**4-MPy**), adsorbed on NRs by exciting at 514 and at 758 nm. **4-MPy** absorbs in the UV (324 nm), hence molecular resonance enhancement of Raman spectra does not occur at 514 nor at 785 nm.

**Fig. 9:**
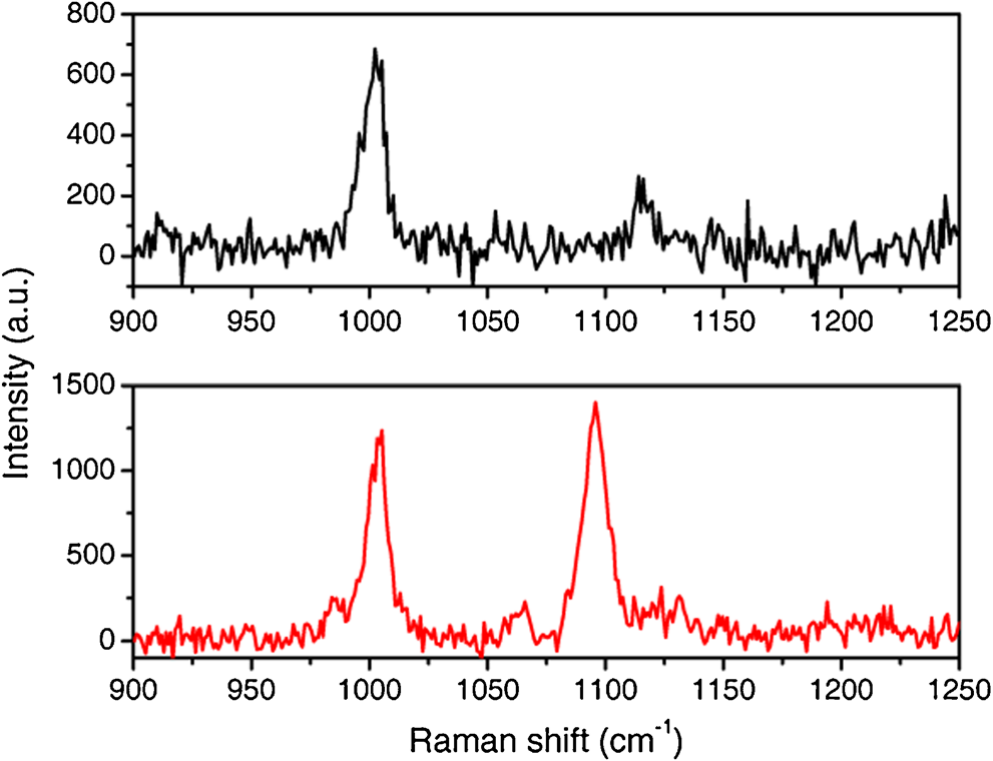
SERS spectra excited at 785 nm, of a 0.022 M aqueous solution of **4-MPy** (*black trace*) and 10^**−**6^ M aqueous solution of **4-MPy** bound to NRs (*red trace*)

A 0.022-M aqueous solution of **4-MPy** was used as a reference for evaluation of the SERS enhancement. Notice that the final analyte concentration in a 2 × 10^12^ particle/mL NRs solution was 10^−6^ M, i.e., almost four orders of magnitude less concentrated than the reference **4-MPy** solution. Aqueous SERS solutions were equilibrated under ambient conditions for 10 min prior to spectral analysis. At 514-nm excitation, no appreciable SERS signal is observed for **4-MPy** bound to NRs (data not shown) due to a small EF, insufficient to compensate for the nano-molar concentration in the NR solution. Conversely, Raman signals of **4-MPy** bound to NRs are strongly amplified when excited by 785-nm laser wavelength (Fig. 9, lower panel).

Characteristic ring breathing modes are observable at 1,004 cm^−1^ (ν(C–C) mode) and at 1,119 cm^−1^ (ν(C–S) mode) in the spectrum of the reference solution (Fig. 9, upper panel). The latter mode is remarkably shifted in **4-MPy** bound to NRs (1,094 cm^−1^) and experiences a dramatic increase in intensity compared with that in the Raman spectrum of the solution. Similar enhancement has been reported for **4-Mpy** adsorbed on other metal substrates, which was interpreted by coordination of **4-Mpy** with the metal surface through the sulfur atom [37].

Notice that, despite the much lower concentration (by almost four orders of magnitude), the SERS spectrum exhibits a better signal-to-noise ratio than the solution one. The evaluated EF for the ν(C–C) ring breathing mode at 1,004 cm^-1^ and the ν(C–S) ring breathing mode at 1,119 cm^−1^ are 3.7 × 10^4^ and 1.1 × 10^5^, respectively, in reasonable agreement with the **PEP+C3-NRs** and **PEP+C12-NRs** measurements. The very fact that an appreciable SERS signal could not be detected when exciting at 514 nm confirms that the EF at resonance with the transverse plasmon is at least one or two orders of magnitude weaker than at resonance with the longitudinal one. Since the normalized intensities defined by R(488, 514) in Table 2 are in the range 3–7, the results reported above for **PEP+C3-NR**s can only be explained considering that resonance with the molecular excitation contributes an enhancement of the Raman signal comparable to that due to resonance with the transverse plasmon.

Finally, Table 2 shows that EFs of **PEP+C3-NRs** and **PEP+C12-NRs** do not exhibit any clear distance dependence although it would be expected in the range of a factor of 2–4 from literature data [38]. We tentatively explain this apparent lack of distance dependence with the following two arguments:

- the packing density of **C3PEP+** and **C12PEP+** is assumed to be equal, but it could be limited in **PEP+C3-NRs** by the bulky heterocyclic heads. In **PEP+C12-NRs** the longer alkyl chains may help achieving a more compact packing.
- the SERS measurements are taken from different samples (the same batch of NRs, a part functionalized with PEP+C3-NRs and a part with PEP+C12-NRs) and therefore reproducibility issues throughout the functionalization/purification processes could contribute to hinder the expected distance dependence.

## Experimental

### Materials

Sodium borohydride (NaBH_4_, ∼99 %), L-ascorbic acid (99 %), hydrogen tetrachloroaurate(III) trihydrate (HAuCl_4_ · 3H_2_O, ≥99.9 %), cetyltrimethylammonium bromide (CTAB), and silver nitrate (AgNO_3_, 99.9999 %) were purchased from Aldrich. Cyclohexane (≥99.5 %) was obtained from Fluka. Acetone (99.8 %) was purchased from Carlo Erba. Stock ion solutions were prepared using deionized water (Millipore milli-Q Gradient A-10 system). NH_4_OH (30 %) was purchased from Fluka. Absolute ethanol was purchased from Baker. Dry acetone was stored over CaCl_2_.

#### Preparation of PEP±C3SAC and PEP±C12SAC

**Fig. 10:**
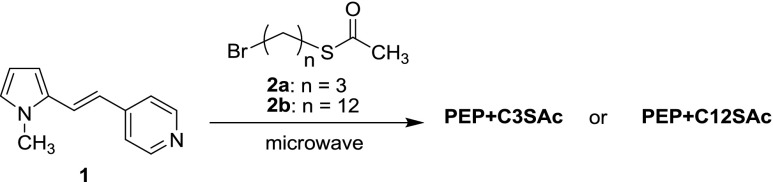
Reaction scheme for the synthesis of PEP+C3SAC and PEP+C12SAC

The two ω-acetylthioalkyl derivatives of **PEP+** were prepared according to Fig. 10 by microwave-promoted alkylation of 1-(pyrid-4-yl)-2-(*N*-methylpyrrol-2-yl)ethylene (**1**)^21^ with the proper ω-acetylthioalkyl bromide **2a** [39] or **2b** [40].

**PEP+C3SAc**. A solution of 1-(pyrid-4-yl)-2-(*N*-methylpyrrol-2-yl)ethylene (**1**) (0.102 g, 0.55 mmol) and ω-acetylthiopropyl bromide (0.218 g, 1.10 mmol) in dry acetonitrile (3 mL) was irradiated at 100 W for 2 h in a microwave reactor. The formed precipitate was collected by filtration to afford the product as a red solid (0.131 g, 0.34 mmol, 62 %); ^1^H-NMR (DMSO-*d*_6_) δ 8.75 (d, *J* 7.0, 2H, pyridine ring), 8.14 (d, *J* 7.0, 2H, pyridine ring), 7.94 (d, *J* 15.9, 1H, vinylene), 7.12 (bs, 1H, pyrrole), 7.08 (d, *J* 15.9, 1H, vinylene), 6.89 (dd, *J* 3.5, 2.2, 1H, pyrrole), 6.24 (dd, *J* 4.1, 3.2, 1H, pyrrole), 4.53 (t, *J* 7.7, 2H, CH_2_N^+^), and 3.80 (s, 3H, NCH_3_); other signals are covered by solvent (2.5 ppm) and water (3.4 ppm) peaks; Anal. Calcd. for C_17_H_21_BrN_2_OS: C, 53.54; H, 5.55; N, 7.35; Found: C, 53.04; H, 5.68; N, 7.22.

**PEP+C12SAc**. A solution of 1-(pyrid-4-yl)-2-(*N*-methylpyrrol-2-yl)ethylene (**1**) (0.184 g, 1.00 mmol) and ω-acetylthiododecyl bromide (0.890 g, 2.75 mmol) in dry acetone (5 mL) was irradiated at 110 W for 3 h in a microwave reactor. After cooling to room temperature the precipitate was filtered and washed with cold acetone, yielding the product as an orange solid (0.300 g, 0.590 mmol, 59 %); ^1^H-NMR (DMSO-*d*_6_) δ 8.80 (d, *J* 6.5, 2H, pyridine ring), 8.18 (d, *J* 6.5, 2H, pyridine ring), 7.90 (d, *J* 15.8, 1H, vinylene), 7.12 (bs, 1H, pyrrole), 7.08 (d, *J* 15.8, 1H, vinylene), 6.90 (d, *J* 3.7, 1H, pyrrole), 6.20 (d, *J* 4.0, 1H, pyrrole), 4.45 (t, *J* 7.2, 2H, CH_2_N^+^), 3.80 (s, 3H, NCH_3_), 2.84 (t, *J* 7.0, 2H, CH_2_S), 2.30 (s, 3H, SAc), 2.0–1.7 (m, 2H, alkyl chain), 1.6–1.4 (m, 2H, alkyl chain), 1.4–1.0 (m, 16H, alkyl chain); Anal. Calcd. for C_26_H_39_BrN_2_OS⋅1/2H_2_O: C, 60.45; H, 7.80; N, 5.42; Found: C, 60.62; H, 7.85; N, 5.47.

#### Preparation of Water Soluble Au NRs

The water soluble surfactant-capped Au NRs with longitudinal plasmon band at 670 nm were prepared by suitable modifications of previously reported seed-mediated growth approach [41, 42]. Such an approach involves two steps. The former concerns the preparation of a seed solution and was performed by mixing at room temperature two freshly prepared solutions of CTAB (0.2 M) and HAuCl_4_ · 3H_2_O (5 × 10^−4^ M). Then 0.6 mL of a 0.01-M ice-cold aqueous solution of NaBH_4_ were added into the aforementioned mixture under vigorous stirring. Within a few seconds the colour of the solution turned from yellow-orange, ascribable to CTAB-Au^3+^ complexes [43], to brown, indicating the formation of Au seeds (i.e., Au NPs smaller than 3 nm). The seed solution was left stirring for 2 h and used within 5 min after stopping the stirring. The latter step involves the growth of such seeds to form Au NRs at room temperature. Three-milliliter growth solution was prepared by mixing CTAB (0.24 mmol), 65 μL of acetone and 45 μL of cyclohexane (able to influence the micelle morphology), AgNO_3_ (0.31 μmol), and further Au precursor (HAuCl_4_ · 3H_2_O, 0.006 mmol). Under gentle mixing, an ascorbic acid solution was added dropwise (ascorbic acid/Au ratio =2) to reduce Au(III) to Au(I) as indicated by the colour of the growth solution which turned from dark yellow (Au(III)) to colourless (Au(I)). At this point, the NR growth was promoted by adding 60 μL of the seed solution and revealed by the appearance of a blue-violet color in a few minutes. In order to prevent the aggregation of Au NRs owing to standard purification by centrifugation procedure, the dye anchoring experiments were performed on the as prepared NR solution without further purification.

#### Anchoring of PEP+ Dyes on Au NRs

The dyes **PEP+C3SAc** and **PEP+C12SAc** are well soluble in ethanol and less soluble in water, especially for the derivative with the longer alkyl chain. The acetyl group prevents the oxidation of the terminal thiol group and it should be removed before the functionalization of Au NRs. In fact, it is well known from the literature that thioacetates react slowly with Au surfaces and organize in a less ordered SAM [44]. In a typical deprotection procedure, 100 μL of NH_4_OH (30 %) was added in a solution of dye in ethanol 10^−3^ M. The as prepared mixture was left to stand for 20 min and then evaporated to dryness by rotavapor [45]. Such precipitate was dispersed in 1 mL of Au NR solution leaving the mixture to stand for 12 h. The dye-NR complex solution was purified upon repeated cycles of dissolution in water and centrifugation (by centrifuge 5430; Eppendorf, at 8,100 rpm, at 25 °C, for 15 min) to wash out unbound surfactant and dye residuals.

### Instrumentation and Calculations

The linear absorption spectra of bare and functionalized Au NRs were recorded using a Varian Cary 5000 UV–Vis–NIR scanning spectrophotometer in the 300–1,200 nm. NMR spectra were recorded on a Bruker AMX-200 and AMX-500 instruments operating at 200 and 500 MHz, respectively. Coupling constants are given in Hz.

TEM investigations have been performed by a Jeol JEM-1011 microscope operating at 100 kV. The specimens have been prepared by depositing a few drops of aqueous NP dispersions onto a carbon-coated copper grid and by allowing the solvent to evaporate. A systematic statistical analysis of NR size distributions was performed on the basis of low-magnification TEM images with the help of Axio Vision software. At least 100 NPs were counted for each sample.

Mid-infrared spectra have been acquired with a Perkin-Elmer Spectrum One Fourier transform infrared (FTIR) spectrometer equipped with a DTGS (deuterated tryglicine sulfate) detector. The spectral resolution used for all experiments was 4 cm^−1^. For attenuated total reflection (ATR) measurements, the internal reflection element (IRE) was a three-bounce, 4 mm diameter diamond microprism. Cast films were prepared directly onto the internal reflection element, by depositing the solution of interest (3–5 μL) on the upper face of the diamond crystal and allowing the solvent to evaporate completely.

Raman scattering experiments were conducted with two different instruments. The first one was a home-built micro-Raman system, based on a Triax-320 ISA spectrograph, equipped with a holographic 1,800 g/mm grating and a CCD detector (Spectrum One ISA Instruments). The excitation source was a Spectra Physics Ar^+^ laser (Stabilite 2017-06S) operating at 488 and 514.5 nm. A Kaiser Optical System holographic notch filter was used to reduce the stray-light level. An Olympus BX 40 optical microscope equipped with a 50×/0.75 objective was optically coupled to the spectrograph. To avoid optical damage to the sample, the power of the exciting radiation was maintained between 0.15 and 0.5 mW. The Raman spectra were recorded with 10 acquisitions, each of 5 s, between 400 and 1,800 cm^−1^ and with an instrumental resolution of about 2 cm^−1^. The second instrument was a Renishaw RM2000 microRaman spectrometer, coupled with a diode laser source emitting at 785 nm. Sample irradiation was accomplished using the 50× microscope objective of a Leica Microscope DMLM. The beam power was about 0.28 mW. Raman scattering was filtered by a double holographic Notch filter system and collected by an air cooled CCD detector. The Raman spectra were recorded with 3 acquisitions, each of 30 s, between 400 and 1,800 cm^−1^. All spectra were calibrated with respect to a silicon wafer at 520 cm^−1^.

For SERS/SERRS measurements, **PEP+** functionalized NRs were either measured in solutions or deposited by drop casting on glass substrates. In order to compare the signal measured at different wavelengths, Raman spectra were normalized by using cyclohexane as an intensity standard following the method of Ref. [46].

#### DDA Calculations

The local field was calculated by the Discrete Dipole Approximation (DDA) method using the software developed by Draine and Flatau (DDSCAT 7.1 and the relative DDFIELD code) [47, 48]. The DDA is one of the most frequently used method to calculate the optical properties and the local field of metal nanostructures of arbitrary shape [49–51]. In DDA, one can account for the structure of interest, usually called “target”, by a cubic array of N polarizable points (i.e. N cubic dipoles). In this work, the target consisted in a cylinder with two hemispheroidal caps, with a main axis of 29 nm and a minor axis of 12 nm (inset in Fig. 2a). We used 173112 dipoles for the target, corresponding to an interdipole spacing of 0.27 nm. For metal particles in the 2–200-nm-size range, an error smaller than 10 % is usually achieved using a number of dipoles at least of the order of 10^4^ and using interdipole spacing much smaller than the wavelengths of interest. The effect of solvent has been accounted for through the refractive index of the non-absorbing matrix, that is *n* = 1.334 for water. The refractive index of water and the Au dielectric constants from Palik were used [52]: we adopted a size-corrected dielectric constant, as reported in ref. [53]. Such dependence is due to the conduction electrons mean free path being comparable to particles size along the direction of polarization promoted by the electromagnetic field. [50, 53, 54]. The size correction has been treated separately for transverse and longitudinal excitation, since the mean free path is different in the two cases. Hence the dielectric constant is anisotropic due to the different size correction applied.

Simulated extinction spectra include the orientational average. The average has been worked out over the three possible orientations corresponding at the three situations in which the three rod axes are, one by one, parallel to the (linearly polarized) incoming beam.

As for the near-field calculations, the incident wavelength was 514 or 785 nm for the electric field parallel to the transverse or longitudinal axis of the rod, respectively. We calculated the square of the electric field by the DDFIELD code and plotted it in the plane that bisects the rod. Near-field was calculated at 0.5 nm from the surface of the NRs.

## Conclusions

In this study, we measured Raman spectra of the heteroaromatic positively charged **PEP+** dyes bound to Au NRs through linear alkyl chains of different lengths (3 or 12 Carbon atoms) terminated with a thiol group. Measurements were realized using the 488, 514, and 785-nm laser excitation lines to study the effect of the laser resonance with the two plasmon modes of NRs and with the absorption band of the molecules.

We observed that the amplification of the Raman signals is higher when the laser excitation wavelength is resonant with the longitudinal plasmon mode. Raman spectra performed exciting at 514-nm are only 4–7 times lower in intensity compared with the 785-nm excitation, indicating that the resonance condition of the 514-nm laser excitation with the absorption band of the dye strongly contributed to the amplification of the Raman signal.

To strengthen the previous experimental evidence we used 4-mercaptopyridine (**4-MPy**) as a standard analyte. Since it absorbs in the UV region, resonance with molecular excitations does not take place at 514 and 785 nm. EF measured at 785 nm turned out to be about 4 · 10^4^ for the ring breathing mode at 1,004 cm^−1^. In addition, the fact that at 514 nm the signal was not detectable confirms that the EF at resonance with the transverse plasmon is at least one order of magnitude lower than the longitudinal one.

In the light of these considerations, the resonance with the absorption band of **PEP+** moiety is the main contribution to the enhancement in the Raman signal of the dye observed at 514-nm laser excitation.

Finally, this work evaluated by SERS the effectiveness of gold NRs as plasmonic enhancers of the optical properties of PEP+ dyes under molecular resonant and non-resonant conditions. Since both SERS and two-photon absorption depend on the forth power of the local electric field, and since **PEP+** dyes are well-known to possess remarkable nonlinear optical properties, the results presented in this paper are also expected to open the way to the improvement of nonlinear optical responses.
